# Visualization of a gallbladder neuroendocrine carcinoma using a novel peroral cholangioscope

**DOI:** 10.1055/a-2098-1350

**Published:** 2023-06-22

**Authors:** Lin Zhou, Yi Wang, Fan Zhou, Muhan Ni, Lei Wang

**Affiliations:** Department of Gastroenterology, Nanjing Drum Tower Hospital, Affiliated Hospital of Nanjing University Medical School, Nanjing, China

Neuroendocrine tumor (NET) of the gallbladder is extremely rare, accounting for less than 1 % of primary gallbladder cancers 1 . Here, we present a case of gallbladder NET in which the tumor was visualized directly using a novel peroral choledochoscope (eyeMax; Micro-Tech, Nanjing, China) with an outer diameter of 2.3 mm.

An 89-year-old man was admitted to our hospital because of a gallbladder mass. Contrast‐enhanced abdominal computed tomography (CT) showed a mass in the gallbladder neck, and endoscopic ultrasound revealed a hypoechoic lesion in this region ( Fig. 1 ). Endoscopic retrograde cholangiopancreatography was performed to confirm the diagnosis of the gallbladder lesion ( Fig. 2 , Video 1 ). Following successful bile duct cannulation, the cystic duct orifice and route were visualized on the cholangiogram. A guidewire and cannulating catheter were inserted into the cystic duct and gallbladder. Bile was collected via the catheter for cytological analysis, and then the cholangioscope was inserted into the gallbladder. Cholangioscopy revealed an irregular elevated lesion with irregularly dilated vessels and papillary characteristics at the gallbladder neck, which was suspected to be a malignant tumor. However, targeted biopsy could not be performed because the forceps could not be passed through the working channel of the cholangioscope.

**Fig. 1 a:**
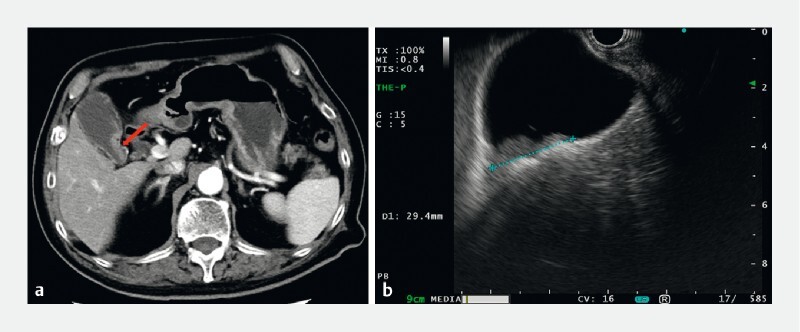
Contrast-enhanced computed tomography showing an enhancing gallbladder mass (arrow). **b** Endoscopic ultrasound showing a hypoechoic lesion at the gallbladder neck.

**Fig. 2:**
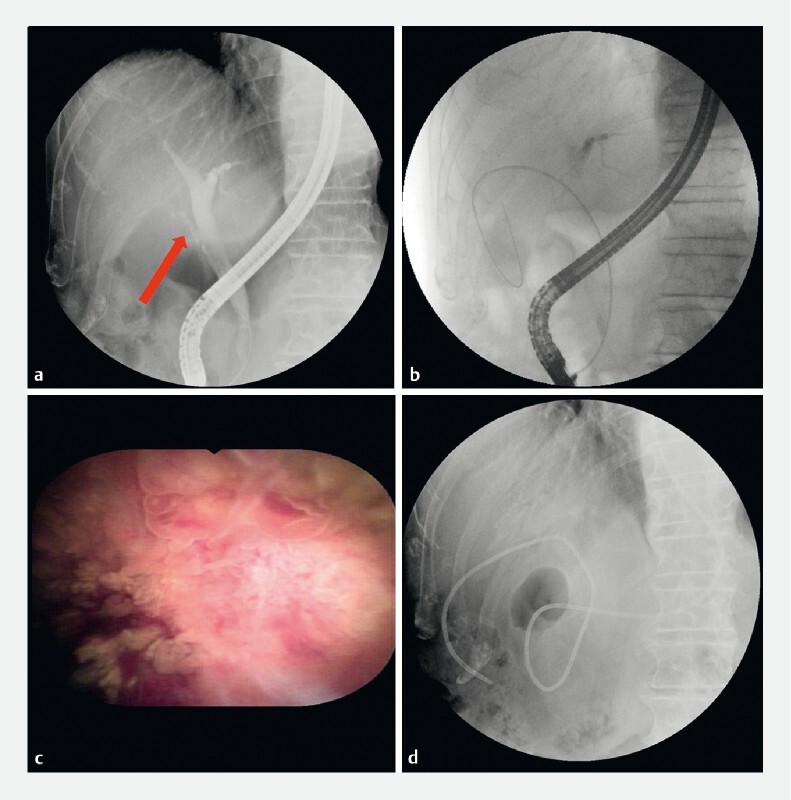
Endoscopic retrograde cholangiopancreatography. **a** Cholangiography showing the cystic duct orifice and route (arrow). **b** Fluoroscopic image showing the guidewire and cannulating catheter inserted into the gallbladder. **c** Visualization of the gallbladder lesion through the peroral choledochoscope. **d** Indwelling nasobiliary tube in the gallbladder.

**Video 1** Insertion of a novel cholangioscope into the gallbladder of an 89-year-old man visualized an irregular elevated lesion, pathologically confirmed to be a neuroendocrine tumor.


Despite negative cytology for malignancy, we recommended surgery based on CT and cholangioscopic findings. Because of the patient’s advanced age, laparoscopic cholecystectomy was performed for the diagnosis and palliative treatment rather than radical cholecystectomy. Interestingly, postoperative pathological analysis confirmed the diagnosis of small-cell NET with positive immunohistochemical staining for synaptophysin, CD56, and pan-cytokeratin (CK) ( Fig. 3 ). The patient survived and currently remains on follow-up.

**Fig. 3 a:**
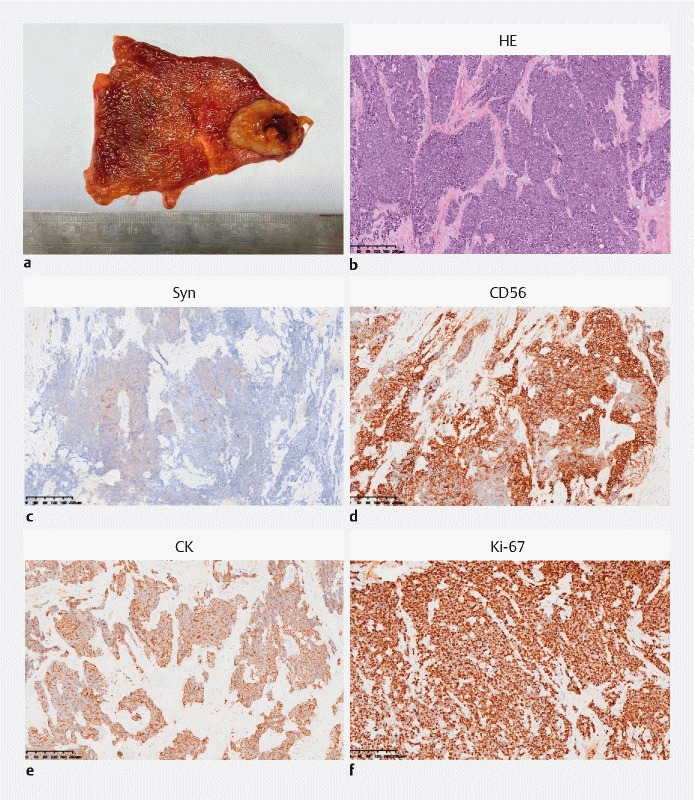
Gross specimen of the malignant tumor. **b** Hematoxylin and eosin (H&E) staining of the tumor specimen. **c–f** Immunohistochemical staining showed that the tumor was positive for synaptophysin (Syn) ( **c** ), CD56 ( **d** ), and pan-cytokeratin (CK) ( **e** ), with a Ki-67 index of 95 % ( **f** ). These findings supported the diagnosis of small-cell neuroendocrine carcinoma.

To the best of our knowledge, this is the first report of visualization of gallbladder NET using a peroral cholangioscope. Although further improvements in the instruments are needed, the use of the eyeMax cholangioscope can be an alternative diagnostic option for gallbladder diseases.

Endoscopy_UCTN_Code_TTT_1AR_2AD

## Notes

Endoscopy E-Videos

https://eref.thieme.de/e-videos
*E-Videos* is an open access online section of the journal *Endoscopy* , reporting on interesting cases and new techniques in gastroenterological endoscopy. All papers include a high-quality video and are published with a Creative Commons CC-BY license. Endoscopy E-Videos qualify for HINARI discounts and waivers and eligibility is automatically checked during the submission process. We grant 100% waivers to articles whose corresponding authors are based in Group A countries and 50% waivers to those who are based in Group B countries as classified by Research4Life (see: https://www.research4life.org/access/eligibility/ ). This section has its own submission website at https://mc.manuscriptcentral.com/e-videos
